# The link between child dietary diversity and child anemia: The power of colorful plates

**DOI:** 10.1371/journal.pgph.0005001

**Published:** 2025-07-30

**Authors:** Amare Abera Tareke, Ahmed Juhar Temam, Addis Alem, Zeleke Geto, Ebrahim Msaye Assefa, Mohammed Derso Bihonegn, Mekonin Belete, Gashaw Abebe, Seid Mohammed Abdu, Altaseb Beyene Kassaw, Gosa Mankelkl, Melese Shenkut Abebe, Hussen Abdu

**Affiliations:** Department of Biomedical Sciences, College of Medicine and Health Sciences, Wollo University, Dessie, Ethiopia; African Population and Health Research Center, KENYA

## Abstract

Nutritional anemias are the most common causes of anemia worldwide, and the extent is greater in the sub-Saharan African (SSA) region. Dietary diversification is among the strategies to prevent anemia due to nutrient deficiencies. There is a lack of evidence on the effect of dietary diversity on anemia, particularly in children. We evaluated the influence of child dietary diversity on anemia among children aged 6–23 months. We used the recent nationally representative cross-sectional surveys from 23 SSA countries. A child with inadequate dietary diversity (treated group) was matched with a child with adequate dietary diversity (control group) based on selected covariates and calculated propensity scores to match 11,742 children in both groups. The effect of child dietary diversity on child anemia was evaluated using independent t-tests, binomial regression, and linear regression. Children with inadequate dietary diversity had a -0.082g/dl (95% CI = -0.137 – -0.027) and p-value < 0.005 lower hemoglobin concentration than children with adequate dietary diversity. Children with inadequate dietary diversity had a 3% increased risk of anemia, compared to children having adequate dietary diversity, RR = 0.97, (95% CI = 0.95 – 0.99) and p-value < 0.05. A one-unit increase in child dietary diversity score was associated with a 0.05 g/dl increase in child hemoglobin (β = 0.05, 95% CI: 0.03 – 0.06), with p-value of < 0.001. One unit increase in dietary diversity score among lower dietary diversity groups resulted in a 0.12 g/dl, (95% CI 0.09 – 0.15g/dl) increase in child hemoglobin level (p-value < 0.001). Inadequate dietary diversity significantly increases the risk of anemia in children. Adequate dietary diversity in children resulted in small but significantly higher mean hemoglobin concentration. To effectively combat anemia in children it is imperative to implement multifaceted interventions that promote dietary diversity and improve food security.

## Introduction

Anemia, characterized by a deficiency in the number or oxygen-carrying capacity of red blood cells [leading to the impaired oxygen delivery to tissues], remains a persistent global health challenge [1,2]. The condition is prevalent among vulnerable populations with marked disparities in age, sex, and geographical areas [2]. The World Health Organization (WHO) estimated that, approximately 1.62 billion people worldwide are affected by anemia [2]. Although anemia prevalence has declined in different segments of the population [3], the progress is insufficient to meet the World Health Assembly global nutrition target [4], and the prevalence of anemia in children also remains high [3]. Anemia has the highest prevalence in sub-Saharan Africa (SSA) and South Asia, especially among children and reproductive-age women. The WHO SSA region is among the most affected regions with an estimated 103 million anemic children [5]. Gender disparities were also noted leaving the females among the risky segment of the population compared to males [2].

Children under five years of age are particularly susceptible to anemia, studies reported a prevalence as high as 65% in African children [6]. This vulnerability is due to increased demand associated with rapid growth, inadequate dietary intake, and increased susceptibility to infection which impairs the absorption and utilization of nutrients including iron [7]. Furthermore, in some regions, cultural practices and socio-economic conditions further exacerbate the nutritional deficiencies that lead to anemia [8]. Genetic blood disorders, such as sickle cell disease, thalassemia, and G6PD deficiency, are common in SSA as well [9]. These factors are crucial as deficiency anemia is the most common form of anemia [2], and is highly prevalent, especially in developing countries [10].

Among the causes of anemia, nutritional deficiency anemias are the most important types of anemia in SSA. Deficiency anemias including those caused by the deficiencies in iron, folate, and vitamin B12 remain the leading causes worldwide [5,11], other causes to a lesser extent include vitamin C, vitamin D, riboflavin, and zinc [7]. Deficiency anemias are linked with inadequate dietary diversity, low socio-economic status, and limited nutrition literacy [12,13]. In SSA iron deficiency is prevalent among children with up to 75% being anemic [14]. Additionally the presence of infectious diseases such as malaria further exacerbates the condition contributing to nutrient deficiencies and then anemia [14,15].

A diversified diet is considered a vital strategy to prevent anemia in children, especially among children aged 6–23 months. A diversified diet enhances the consumption of essential micronutrients such as iron, vitamin A, and folate, which are vital for hematopoiesis and overall growth [16,17]. In addition, intake of a diversified diet can enhance the overall nutritional status of children, promoting growth and development. Moreover, the timing of the diversified complementary food is critical. At the age of 6 months, the iron store of the child is depleting, and the maternal supply from milk is not enough and therefore exposes the child to anemia [18].

While ample of previous studies focused on maternal dietary diversity and child anemia, only a few studies in this region [19] and elsewhere [20] evaluated the association between child dietary diversity and anemia. These studies were cross sectional unable to infer causality between child dietary diversity and child anemia. In this study, we matched children on various variables affecting treatment assignment and created pseudo-randomization. It simulated experimental design (quasi-experimental) and infer causality on the effect of child dietary diversity and anemia. The study, thus, provides stronger evidence for causal relationship dietary diversity and child anemia.

## Methods

### Ethics statement

The DHS data is publicly available after the removal of all personal identifiers of study participants. Procedures and questionnaires for standard DHS surveys have been reviewed and approved by ICF Institutional Review Board (IRB). Additionally, country-specific DHS survey protocols were reviewed by the ICF IRB and typically by an IRB in the host country. ICF IRB ensured that the survey complies with the U.S. Department of Health and Human Services regulations for the protection of human subjects (45 CFR 46), while the host country IRB ensured that the survey complies with the laws and norms of the nation. We used the publicly available data for analysis, permission to analyze the data was granted by the DHS program Office.

### Setting and data source

This study used Demographic and Health Survey (DHS) data from 23 SSA countries with data on child hemoglobin and dietary diversity. The countries included are Angola, Burkina Faso, Benin, Burundi, Cote d’Ivoire, Cameroon, Ethiopia, Gabon, Ghana, Gambia, Guinea, Liberia, Madagascar, Mali, Mauritania, Malawi, Mozambique, Nigeria, Rwanda, Sierra Leone, South Africa, Zambia, and Zimbabwe. The data used for this study were downloaded from MEASURE DHS (https://dhsprogram.com/data/available-datasets.cfm) after obtaining permission to access the datasets. We used Kids Recode (KR) files from each country. Data was accessed on May 8^th^ 2024. The authors of this study did not have access to information that could identify individual participants during or after data collection.

### Population

We were interested in evaluating the role of dietary diversity on child anemia. To this end, our target population was children 6–23 months of age. We included children in this age group if they were single births (multiple births are excluded), and there is hemoglobin measurement. Since dietary diversity was our treatment variable, missing this variable led to exclusion. With the same scenario missing the outcome variable (child hemoglobin) was the exclusion criteria as well. We selected the last-born child and kept children aged 6–23 months for further analysis. The number of children included from each country, survey year, and the sample size are outlined in Table 1.

**Table 1:** The number of children aged 6-23 months included in the current analysis.

			Before matching		After matching	
	Country	Year	Frequency	Percent	Frequency	Percent
1	Angola	2016	2057	5.34	745	6.34
2	Burkina Faso	2018	1733	4.50	427	3.64
3	Benin	2018	2044	5.31	712	6.06
4	Burundi	2017	1938	5.04	675	5.75
5	Cote d’Ivoire	2021	1535	3.99	387	3.3
6	Cameroon	2018	1402	3.64	506	4.31
7	Ethiopia	2016	2779	7.22	598	5.09
8	Gabon	2020	1774	4.61	693	5.9
9	Ghana	2022	1477	3.84	622	5.3
10	Gambia	2020	1186	3.08	328	2.79
11	Guinea	2018	1045	2.71	248	2.11
12	Liberia	2020	858	2.23	157	1.34
13	Madagascar	2021	1829	4.75	513	4.37
14	Mali	2018	1365	3.55	347	2.96
15	Mauritania	2021	1529	3.97	411	3.5
16	Malawi	2016	1616	4.20	420	3.58
17	Mozambique	2023	1215	3.16	293	2.5
18	Nigeria	2018	3634	9.44	1353	11.52
19	Rwanda	2020	1184	3.08	389	3.31
20	Sierra Leone	2019	1454	3.78	444	3.78
21	South Africa	2016	424	1.10	114	0.97
22	Zambia	2018	2803	7.28	825	7.03
23	Zimbabwe	2015	1609	4.18	535	4.56
	Total		38490	100	11742	100

### The treatment

Our treatment variable was dietary diversity, and defined according to DHS recommendations [21]. Dietary diversity (minimum dietary diversity) indicates the percentage of children aged 6–23 months who were living with their mother and were fed at least five out of the following eight food groups during the previous night. The food groups are 1) breastmilk, 2) Grains, white/pale starchy roots, tubers, and plantains, 3) Legumes and nuts, 4) Dairy products (infant formula, milk, yogurt, and cheese), 5) Flesh foods (meat, fish, poultry, and liver/organ meats), 6) Eggs, 7) Vitamin A rich fruits and vegetables, and 8) Other fruits and vegetables.

### The outcome

Our outcome variable was child anemia status. Anemia in children is defined as hemoglobin level below 11g/dl. For descriptive statistics mild anemia is a hemoglobin level between 10.0-10.9g/dl, moderate is between 7.0 and 9.9g/dl and severe anemia is below 7.0g/dl [22].

### Variables

Variables for matching were selected based on their role in the probability of the child having a diversified diet or not. If the variable affects child dietary diversity (probability of treatment) or the standardized difference is relatively large the variable was used as a matching variable. The matching variables were maternal education (No education, Primary, Secondary, and Higher), country, number of household members (<7 and>=7 based on mean), birth weight (low birth weight or otherwise), place of residence (urban and rural), maternal age (standard 5-year interval), currently pregnant or not, union status (not in a union, currently in a union, or formerly in a union), if the mother was working during the time od survey or not, child age, sex of the household head (male vs female), wealth index (Poorest, Poorer, Middle, Richer, and Richest)). Propensity scores were generated by a binary logistic regression model to compute the probability of a diversified diet. Up on binary logistic regression, the above variables were related to the treatment variable and selected as covariates [23].

### Statistical analysis

The DTA file of the 23 countries was downloaded, and all analyses were performed using Stata Version 17.0. Descriptive statistics were used to describe participants before and after matching.

### Propensity score analysis

Since DHS data is cross-sectional in nature, causal inference is difficult without statistical adjustments. To overcome this inherent deficiency, we applied propensity score matching. The propensity score matching allows analysis of observational data by accounting for the randomization issue using baseline variables [24]. The propensity score, thus, is the probability of treatment assignment conditional on observed baseline characteristics. The method allows to design and analyze observational studies so that they mimic characteristics of a randomized controlled trial [24]. It is a balancing score conditional on the propensity score, the distribution of observed covariates will be similar between treated and untreated subjects. Child pairs were formed, such that matched children had similar values of propensity scores. Thus, for a given child in a certain arm, all the subjects from the other group whose propensity score lay within a specified distance (caliper) were identified. The smaller distance with a set of covariates corresponds better matching. We used a caliper distance of 0.01 considering this notion. If no child had a propensity score that matches a given child from the other group, that child was removed from the matched sample. Standardized differences [25] were calculated to compare child features before and after matching with imbalance being defined as an absolute value greater than 0.10 [24].

After generating the propensity score nearest neighbor matching was used with a caliper of 0.01 with a 1:1 ratio of treatment and controls. Child dietary diversity was matched with a sample of 5871 in each group, with a total of 11742 children.

Matched frequencies and percentages were calculated. The mean hemoglobin concentration was calculated for treatment and control groups using an independent t-test. The association between dietary diversity and child anemia was computed by binomial regression, relative risk (RR) with 95% confidence interval was also calculated for dietary diversity and child anemia. Since our data is a quasi-experimental data after the statistical treatment, and we were interested in computing RR rather than odds ratio, we used binomial regression. Mean differences from respective mean hemoglobin concentrations were also computed, and the statistical significance was sought by independent t-test. To delineate the association between dietary diversity score and child hemoglobin linear regression was performed using dietary diversity score as an independent variable with a continuous scale. Statistical significance was declared using a P value less than or equal to 0.05 for all analyses.

## Results

We used 38490 and 11742 unmatched and matched children aged 6–23 months from 23 SSA countries. The description of the sample before and after matching is provided in Table 2. Apart from the description based on several socio-demographic variables, Table 2 shows the standardized difference before and after matching. For example, regarding maternal education, the “No education” group was 39.2% and 24.7% among inadequate and adequate dietary diversity groups before matching. After matching this percentage becomes 24.6% and 25.1%, the standardized difference also decreased to 0.086 after matching.

**Table 2:** Characteristic of matched and unmatched populations, the standardized mean difference before and after, frequencies and percentages.

Characteristics	Unmatched			Matched		
	Inadequate N = 32,264	Adequate N = 6,226	Difference	Inadequate N = 5,871	Adequate N = 5,871	Difference
Maternal education						
No education	12653 (39.2)	1536 (24.7)	0.49620	1445 (24.6)	1474 (25.1)	0.08641
Primary	10280 (31.9)	1521 (24.4)		1665 (28.4)	1468 (25.0)	
Secondary	8488 (26.3)	2574 (41.3)		2329 (39.7)	2411 (41.1)	
Higher	843 (2.6)	595 (9.6)		432 (7.4)	518 (8.8)	
Maternal Age						
15-19	3256 (10.1)	434 (7.0)	0.13754	456 (7.8)	407 (6.9)	0.04916
20-24	8113 (25.1)	1442 (23.2)		1394 (23.7)	1371 (23.4)	
25-29	8204 (25.4)	1706 (27.4)		1583 (27.0)	1608 (27.4)	
30-34	6217 (19.3)	1355 (21.8)		1287 (21.9)	1265 (21.5)	
35-39	4315 (13.4)	889 (14.3)		798 (13.6)	842 (14.3)	
40-44	1754 (5.4)	339 (5.4)		285 (4.9)	319 (5.4)	
45-49	405 (1.3)	61 (1.0)		68 (1.2)	59 (1.0)	
Members	6.98 (4.47)	6.64 (4.29)	0.07877	6.82 (4.79)	6.68 (4.34)	0.03063
Residence (Urban)	9951 (30.8)	3186 (51.2)	0.42246	2875 (49.0)	2951 (50.3)	0.02589
LBW	1916 (5.9)	413 (6.6)	0.02864	394 (6.7)	385 (6.6)	0.00616
Wealth index						
Poorest	8885 (27.5)	892 (14.3)	0.53470	890 (15.2)	865 (14.7)	0.06813
Poorer	7488 (23.2)	974 (15.6)		938 (16.0)	929 (15.8)	
Middle	6657 (20.6)	1168 (18.8)		1191 (20.3)	1122 (19.1)	
Richer	5193 (16.1)	1372 (22.0)		1356 (23.1)	1287 (21.9)	
Richest	4041 (12.5)	1820 (29.2)		1496 (25.5)	1668 (28.4)	
Child age (months)						
6-8	6409 (19.9)	353 (5.7)	0.50517	472 (8.0)	332 (5.7)	0.09567
9-11	5708 (17.7)	722 (11.6)		694 (11.8)	684 (11.7)	
12-23	20147 (62.4)	5151 (82.7)		4705 (80.1)	4855 (82.7)	
Pregnant	1304 (4.0)	624 (10.0)	0.23553	539 (9.2)	560 (9.5)	0.01228
Household head (male)	25142 (77.9)	4754 (76.4)	0.03736	4510 (76.8)	4496 (76.6)	0.00564
Union status						
Never in union	2740 (8.5)	588 (9.4)	0.04953	540 (9.2)	551 (9.4)	0.01501
Currently	27683 (85.8)	5337 (85.7)		5025 (85.6)	5032 (85.7)	
Formerly	1841 (5.7)	301 (4.8)		306 (5.2)	288 (4.9)	
Currently working	18559 (57.5)	3987 (64.0)	0.13375	3706 (63.1)	3762 (64.1)	0.01983

### The effect of child dietary diversity on child anemia

Our results from the binomial regression indicated that adequate dietary diversity reduces the risk of anemia among children aged 6–23 months. The risk of anemia was reduced by 3%, RR = 0.97, 95% CI (0.95 - 0.99), and p-value = 0.035. We also compared the mean hemoglobin concentration of children with and without adequate dietary diversity after matching. We found that children with adequate dietary diversity have significantly higher hemoglobin concentration than children having inadequate dietary diversity [Table 3]. The mean hemoglobin concentration of children with inadequate dietary diversity is 10.20g/dl and those with adequate dietary diversity are 10.27g/dl. This corresponds to a mean difference of -0.082g/dl, although small, the findings depicted a statistically significant reduction in hemoglobin level associated with inadequate dietary diversity (p-value = 0.0033).

**Table 3:** Independent sample t-test showing the mean difference in hemoglobin concentration between children with and without adequate dietary diversity.

Groups	Observation	Mean	SE	SD	LB	UB
Inadequate	5,871	10.18951	0.0200313	1.534845	10.15024	10.22878
Adequate	5,871	10.27174	0.0195001	1.494144	10.23352	10.30997
Combined	11,742	10.23063	0.0139823	1.515125	10.20322	10.25803
Difference		-0.0822347	0.0279554		-0.137032	-0.0274375

SE: Standard error, SD: Standard deviation, LB: lower 95% confidence interval, UB: Upper 95% confidence interval.

In Table 3, the “inadequate” and “adequate” represents children with inadequate and adequate dietary diversity respectively. The “combined” represents the two groups together. Mean hemoglobin concentration and 95% confidence intervals are provided for both control and treatment groups, as well as the combined. The “difference” represents the mean hemoglobin difference between the two groups.

**Fig 1:**
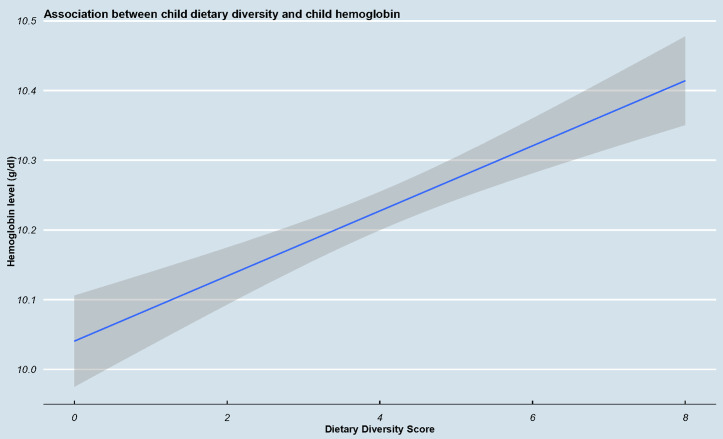
Association between dietary diversity score and child hemoglobin. There is direct positive relationship between increased dietary diversity score and child hemoglobin. As shown in the figure, the mean hemoglobin concentrations of children tend to be highest when the dietary diversity approaches the maximum of 8 food groups.

We also conducted linear regression analysis on the matched sample to assess the relationship between child hemoglobin and child dietary diversity score as continuous variables. We found that a 1 unit increase in child dietary diversity score was associated with a 0.05 g/dl increase in child hemoglobin (β = 0.05, 95% CI: 0.03 – 0.06), with a statistically significant p-value (p < 0.001) (Fig 1).

**Fig 2:**
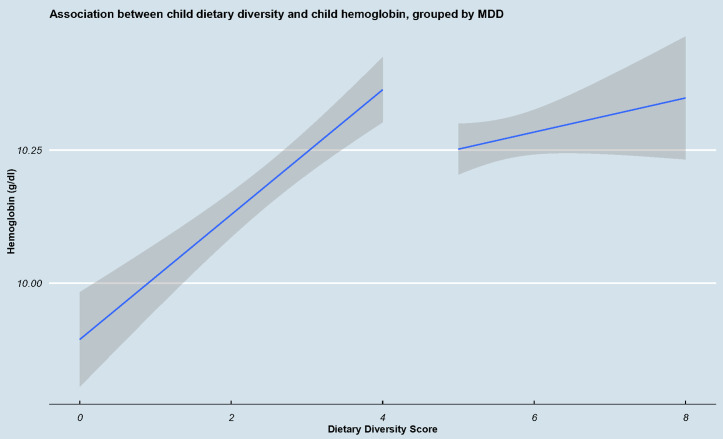
Association between child dietary diversity stratified as adequate and inadequate and child anemia. As shown in Fig 2, as dietary diversity increased, especially among children consumed ≤4 food groups, the regression slope is steep and significant. In contrast, among children with higher dietary diversity, the slope is flatter and it is not statistically significant. This suggests that dietary interventions to avert anemia are more important for inadequate dietary diversity groups.

We also evaluated the association between child hemoglobin level and dietary diversity score among the adequate and inadequate dietary diversity groups. As shown in Fig 2, dietary diversity score has a pronounced impact on anemia among children having inadequate dietary diversity. Specifically, one unit increase in dietary diversity score among this group resulted in a 0.12g/dl, (95% CI 0.09 – 0.15g/dl) increase in child hemoglobin level (p-value < 0.001). Among children with adequate dietary diversity, although an increase in dietary diversity resulted in increased child hemoglobin (Fig 2), the association was not statistically significant (p-value> 0.05).

## Discussion

In recent years the relationship between dietary diversity and anemia has drawn significant attention, particularly in low-resource settings, where a diverse diet is limited. We synthesized evidence in this study using children aged 6–23 months from 23 SSA countries. We applied a propensity score matching to balance covariates between the exposed (inadequate dietary diversity) and unexposed (adequate dietary diversity) groups. We found that inadequate dietary diversity increased the risk of anemia in these children. Increasing dietary diversity score was associated with a significant increase in the child’s hemoglobin level. Although our results showed small effect sizes, they were significant and clinically important. First, the mean difference is due to the effect of dietary diversity alone after balancing covariates. The existence of other factors [26] affecting hemoglobin concentration in the resource-limited settings of SSA may further exacerbate the situation. Second, our population consists of children aged 6–23 months, a group in their critical windows of growth including brain development [27]. Third, lower hemoglobin levels are linked other consequences [28] that might lead to further deterioration of hemoglobin initiating vicious cycle.

Dietary diversity with its deficiencies is critical in assessing nutritional intake and nutrient coverage, particularly in young children. As it measures the number of food groups consumed over 24 hours it serves as a proxy for overall diet quality and nutrient adequacy [29,30]. In the context of anemia and malnutrition more broadly, observational studies consistently showed children with inadequate dietary diversity have higher odds for anemia and other forms of malnutrition [19,31,32]. These findings underscore the importance of a diversified diet in the prevention of not only anemia, but also other forms of malnutrition in children. The findings of this study align with the wider body of evidence [33–35] indicating that dietary diversity is crucial for micronutrient intake including iron and others that are essential for maintaining normal hemoglobin levels. This relationship is especially important in SSA where food insecurity and limited access to a variety of food groups exacerbate nutritional deficiencies. Children aged 6–23 months are typically breastfeeding. Breast milk is low in iron, making iron-rich complementary foods mandatory after six months. Micronutrient deficiencies, particularly iron deficiency are a major cause of anemia worldwide especially in SSA. Dietary diversity is crucial for meeting children’s nutrient needs, including iron and zinc. Breastfeeding mothers may not be aware of the importance of adequate complementary feeding. Cereals, roots, and tubers, common in poor populations, are low in bioavailable iron and can contribute to anemia. Breastfeeding children are more likely to be anemic due to inadequate complementary feeding, highlighting the need for a balanced approach to nutrition [36].

This study uses multi-country data to increase the sample size and provide better matching of control groups. Given the inherent nature of the analysis method to remove unmatched subjects from the analysis, the use of a larger sample is crucial. The selection of children aged 6–23 months for the analysis provides a better picture of the association between dietary diversity and child anemia. These children are under rapid growth and development under the influence of both maternal physiology (through breast milk) and the external environment. While nutrients from breastfeeding including iron are crucial for optimal health and growth, the external environment, including access to diverse food groups and overall diet quality impacts child nutrition in this age group.

The use of propensity score matching provides several advantages over conventional multivariable regression. The method allows the researcher to model the effect of covariates on treatment, rather than modeling both covariates and treatment directly on the outcome. The use of propensity score also balances covariates between treatment and control groups enabling more accurate estimations of the effect of treatment on the outcome [37]. This approach minimizes biases including selection bias. Our use of multiple analytic methods to quantify the effect increases the credibility of results [38].

Despite its strengths, this study has several limitations. The use of propensity score matching despite providing some strengths, has drawbacks as well. The use of this method is associated with possible data loss. In our study, the data loss was not pronounced in the treatment group (~355 subjects removed from the analysis). In contrast, over 26,000 subjects were excluded from the control group, which is expected, given the imbalance in the percentage of children having adequate and inadequate dietary diversity, and we used 1:1 matching. On the other hand, hidden bias is one of the limitations of propensity score matching. To check the analysis method’s reliability in this regard we conducted sensitivity analysis using the Mantel-Haenzel (MH) test statistics. The results [Not Shown] indicated that as gamma increases the probability of underestimating the effect of dietary diversity on child anemia increases, and the probability of overestimation was not significant as gamma increases. This underpins the fact that there might be an underestimation of the treatment effect due to hidden bias.

The implications of these findings are profound, as they highlight the need for comprehensive strategies to enhance the dietary diversity of children in SSA. Interventions may include community-based nutrition education programs that emphasize the importance of diverse diets and provide practical guidance on incorporating a variety of food groups into meals, creating colorful plates. Community based nutrition education for child caregivers [39], nutrition education linked to agricultural interventions [40], and nutrition sensitive agricultural interventions [41] showed significant positive impact to improve dietary diversity in developing countries. Additionally, policies aimed at improving food security and improve access to nutritious foods are essential for fostering an environment where dietary diversity can thrive.

In conclusion, inadequate dietary diversity significantly increases the risk of anemia in children. Adequate dietary diversity in children resulted in higher mean hemoglobin concentration. Furthermore, a unit increase in dietary diversity score is associated with a significant increase in hemoglobin level especially among children with inadequate dietary diversity. To effectively combat anemia in children it is imperative to implement multifaceted interventions that promote dietary diversity and improve food security, especially for lower dietary diversity groups. Such efforts will not only avert deficiency anemia but also contribute to the broader goal of reducing malnutrition in children.
